# Transperineal prostate biopsy: analysis of a uniform core sampling pattern that yields data on tumor volume limits in negative biopsies

**DOI:** 10.1186/1742-4682-7-23

**Published:** 2010-06-17

**Authors:** Gordon R Kepner, Jeremy V Kepner

**Affiliations:** 1 Membrane Studies Project, Minneapolis, Minnesota, USA; 2 Computer Science and Artificial Intelligence Laboratory, Massachusetts Institute of Technology, Cambridge, Massachusetts, USA

## Abstract

**Background:**

Analyze an approach to distributing transperineal prostate biopsy cores that yields data on the volume of a tumor that might be present when the biopsy is negative, and also increases detection efficiency.

**Methods:**

Basic principles of sampling and probability theory are employed to analyze a transperineal biopsy pattern that uses evenly-spaced parallel cores in order to extract quantitative data on the volume of a small spherical tumor that could potentially be present, even though the biopsy did not detect it, i.e., negative biopsy.

**Results:**

This approach to distributing biopsy cores provides data for the upper limit on the volume of a small, spherical tumor that might be present, and the probability of smaller volumes, when biopsies are negative and provides a quantitative basis for evaluating the effectiveness of different core spacing distances.

**Conclusions:**

Distributing transperineal biopsy cores so they are evenly spaced provides a means to calculate the probability that a tumor of given volume could be present when the biopsy is negative, and can improve detection efficiency.

## Background

While transrectal continues to be the predominant prostate biopsy approach, there is increasing interest in the transperineal approach--either initially, or following a negative transrectal biopsy. Biopsy results are categorized as all or none, either a tumor is found, or not--which is the most likely outcome [1]. Rebiopsies bring additional cost and stress. Given the frequency of negative biopsies, it is assumed there would be interest in examining the question: how can a negative transperineal biopsy extract quantitative information about the potential presence of an undetected tumor volume? This theoretical analysis will demonstrate the utility of adapting current transperineal biopsy protocols to one that uses uniformly distributed parallel cores. It is shown that such a protocol increases the efficiency of detecting tumors. Further, if the biopsy is negative, this approach yields quantitative data that sets limits on the volume for small spherical tumors that might be present, but undetected. This is of value because tumor volume is a factor in evaluating the potential for a clinically significant cancer to be present. It can help to reduce the over treating of small cancers.

To our knowledge, no prostate biopsy protocol in current use (transperineal or transrectal) has shown how to obtain quantitative data on tumor volume -- whether the biopsy is positive or negative. The analysis supports further investigation of this alternative to the random systematic protocols for transperineal biopsy. These currently offer no basis for quantitative evaluation of the tumor volume, even if detected by a biopsy core. For positive biopsy results, the key issue becomes the Gleason grade of the tumor sample, which strongly influences the next clinical decision.

## Methods

Mathematical modeling of prostate biopsies has supported the basic principle that more cores can increase the tumor detection probability [2-4]. (For an alternative perspective, see [5,6].) These models did not address the question of what information might be obtained from a negative biopsy. An analytic approach to systematic transperineal biopsy is presented. It assumes, for example, a suitable brachytherapy template and ultrasound guidance are used to deploy a uniform grid of evenly-spaced parallel cores [7]. A recent computer-simulated study of transperineal biopsy described use of a grid pattern of evenly-spaced cores to detect tumor [8]. That idea is extended here by a mathematical analysis that shows how such a pattern enables one to calculate the probability that a small spherical tumor could still be present when biopsies are negative. This mathematical analysis of the transperineal technique relies on a model of the biopsy cores and a model of the tumor.

The biopsy core model employs a grid pattern of evenly-spaced transperineal point cores, shown perpendicular to the transverse cross-section. (The analysis also considers the case of a finite core with radius *R*_c_; see Appendix.) One key parameter of the model is the spacing between the cores, *S*, measured in cm. Depending on prostate size, and the effective cutting length of the biopsy needle, it could require two biopsy cores, stacked end to end, to sample adequately a grid point along a length from apex to base [8]. Because the analysis focuses on detecting smaller tumor volumes, they are modeled as spheres--as others have done [2-4]. The other key parameter is therefore the tumor diameter, *D*_T_, in cm. Define the ratio of these parameters as *n *≡ tumor diameter/core spacing = *D*_T_/*S*.

**Figure 1:**
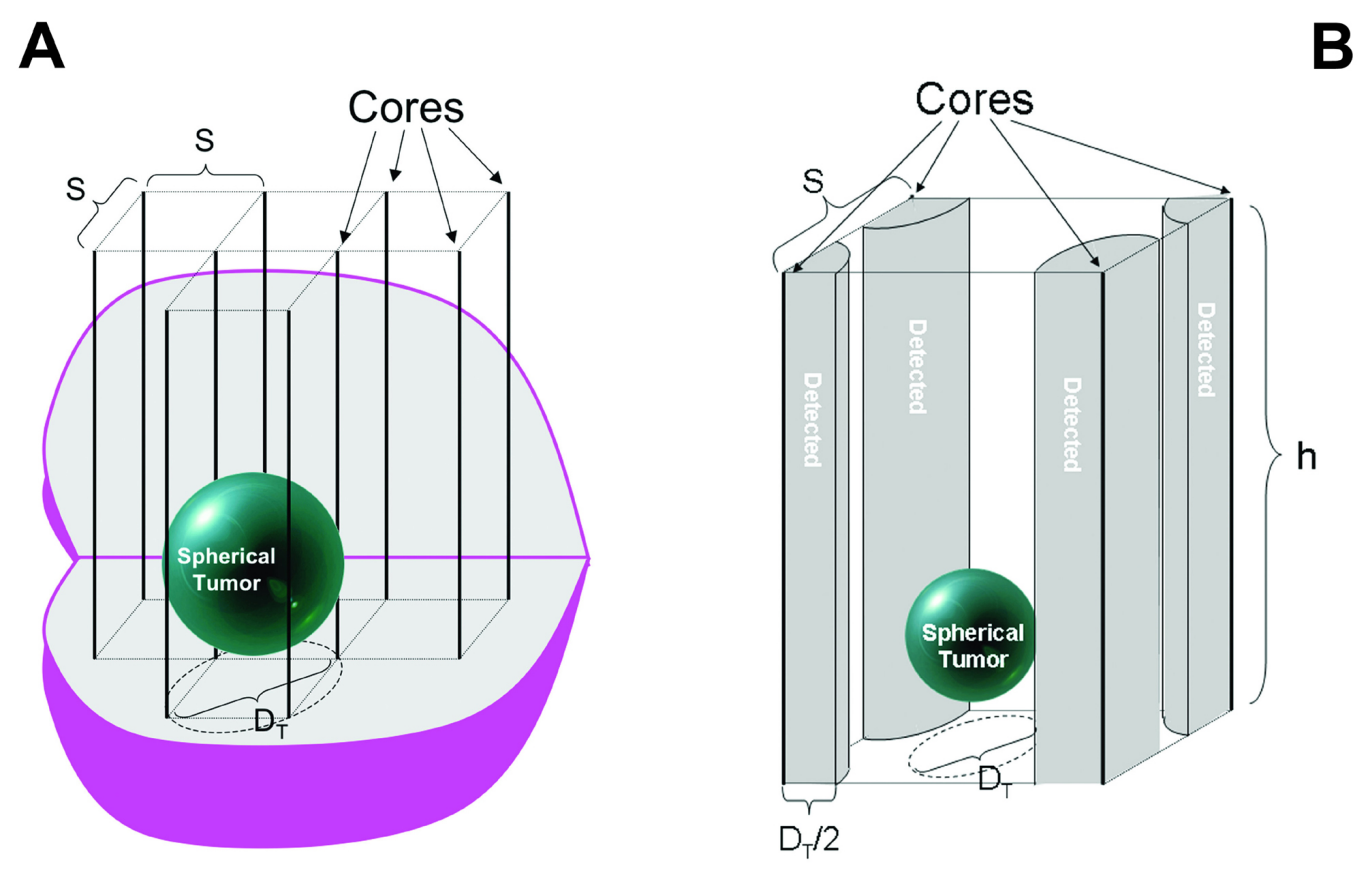
**A. A 3D cutout view of a prostate showing the maximum spherical tumor that can avoid detection in a uniform grid of cores with spacing, *S*, between core centers**. **B**. The grey quarter-cylinders denote the volume in which a small spherical tumor of diameter *D*_*T *_would be detected.

Combining the models, one can estimate the largest tumor that could fit between the biopsy cores and avoid detection (see Figure 1A). The diameter of the largest undetected spherical tumor is related to the core spacing. The largest tumor that fits between the biopsy cores can do so only if its center lies exactly halfway between the cores. Smaller tumors are harder to detect because there are more places where they might lie between the cores. Figure 1B illustrates the locations where the center of a spherical tumor might be detected, versus undetected. If the tumor center is within *D*_T_/2 of a biopsy core, the tumor will intersect the core and be detected. As tumor diameter increases, the effective detection volume of the quarter-cylinders also expands, thereby reducing the available volume wherein the tumor can lie undetected. The mathematical analysis of this uniform transperineal core pattern calculates the probability that a spherical tumor of a given diameter and volume will be detected, based on the ratio of the volume of the locations where it would be detected to the total volume between the cores (see Appendix). If this biopsy pattern yields a positive core, it does not, however, enable one to quantitate tumor volume or tumor volume probabilities.

Note that this analysis is independent of the relative frequency of tumor distribution within the various zones of the prostate. The uniform grid of cores is searching for tumor volume. It does not matter if the tumor volumes are distributed preferentially in one zone or another, because the analysis provides a detection probability value for all tumors of a given volume, regardless of where they reside. The uniform core spacing also maximizes this detection efficiency for each core. Note that a negative biopsy doesn't indicate either tumor absence or tumor presence. Essentially, a negative biopsy establishes nothing certain; it is indeterminate as to the presence of tumor. No current biopsy protocol, of which we are aware, produces a quantitative value for tumor volume in the event of a positive biopsy. The analysis here shows how a redesigned transperineal biopsy protocol can yield quantitative data on tumor volume when the biopsy is negative.

## Results

**Figure 2:**
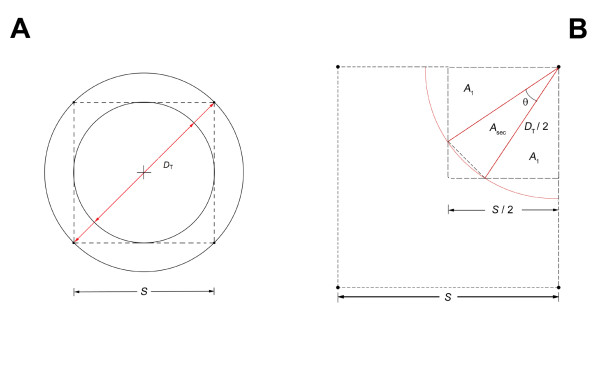
**A. Four evenly-spaced point cores with *S *cm spacing between cores**. The circumscribed circle depicts that largest spherical tumor, of diameter *D*_*T *_= *S*, that just grazes the point core. These contact points form a detection square, with sides *S*. **B**. Point core detection quadrant geometry, where *D*_*T*_/2 is the radius of a generalized tumor, superimposed on this detection quadrant.

Figure 1A and Figures 2A and 2B clarify the relation between a uniformly-spaced grid of point cores and the largest spherical tumor volume that could, potentially, go undetected by the biopsy cores. It has been implied that the core spacing, *S*, defines the diameter of this tumor [9,10]. In fact, as shown by the circumscribed circle in Figure 2A, it is *S *= *D*_T _that defines this spherical tumor's diameter. Thus, *V*_T _= (0.5236) ( S)^3 ^is significantly larger than *V*_T _= (0.5236) *S*^3^, by a factor of 2.8.

**Figure 3:**
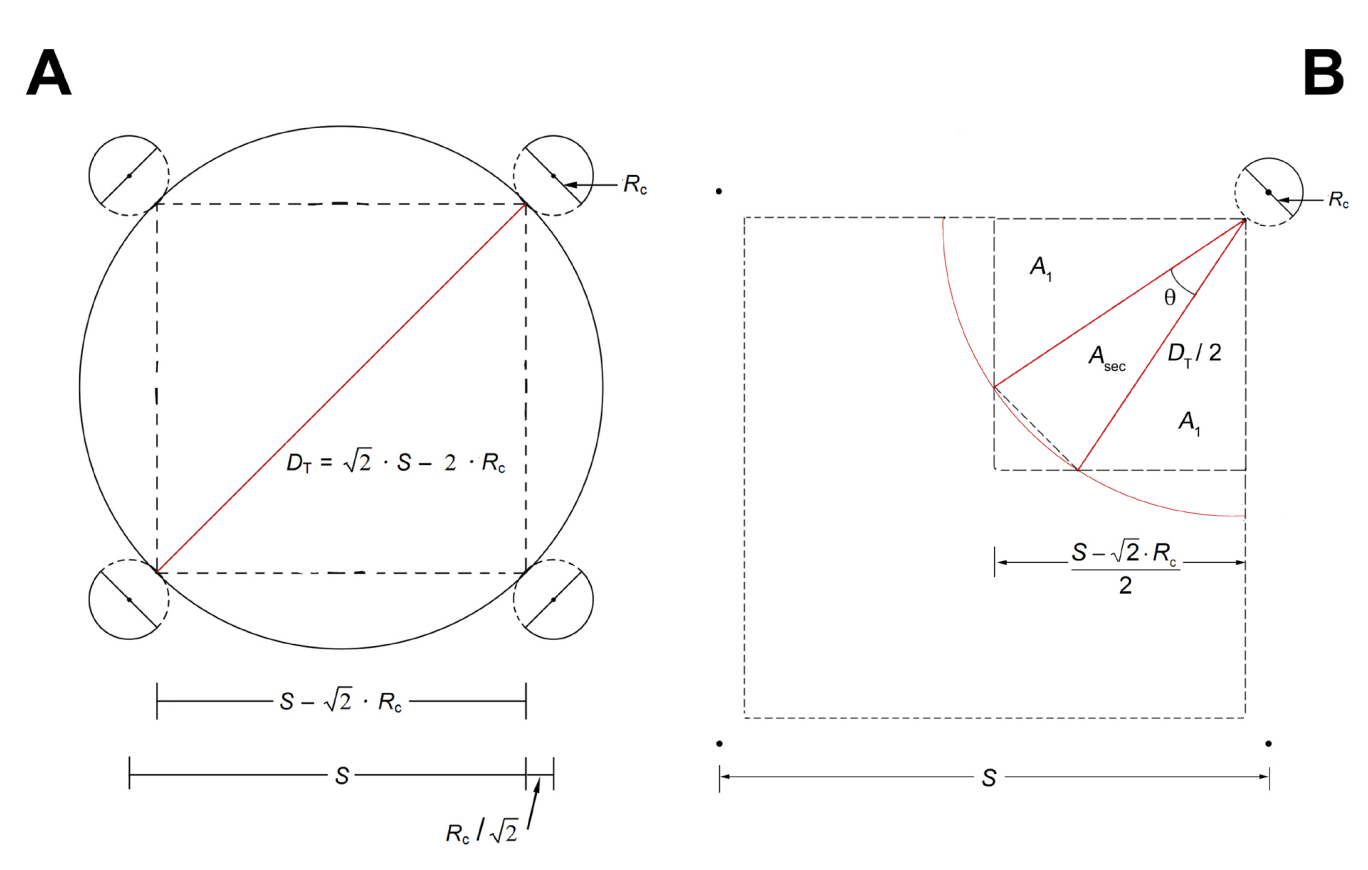
**A. Four evenly-spaced finite cores (not to scale), of radius R_*c*_, where the spherical tumor contact points with the edges of the finite cores form a detection square with sides, *s *≡ *S *- *R*_*c*_**. **B**. Finite core detection geometry where *s *≡ *S *- *R*_*c*_.

The probability of detection (PoD) is given by Appendix equation A1, rewritten here using the effective core spacing for a finite core, *s *≡ *S *- *R*_c_. Then set *n *= *D*_T_/*s *= tumor diameter/effective core spacing (see Figures 3A and 3B). When *R*_c _is zero, point core, then *s *≡ *S *gives the point core case, and now *n *= *D*_T_/*S*. Thus, for *n *≤ 1, then *D*_T_/*s *≤ 1,(1)

When *n *≥ 1, see equation A7,(2)(3)

**Figure 4:**
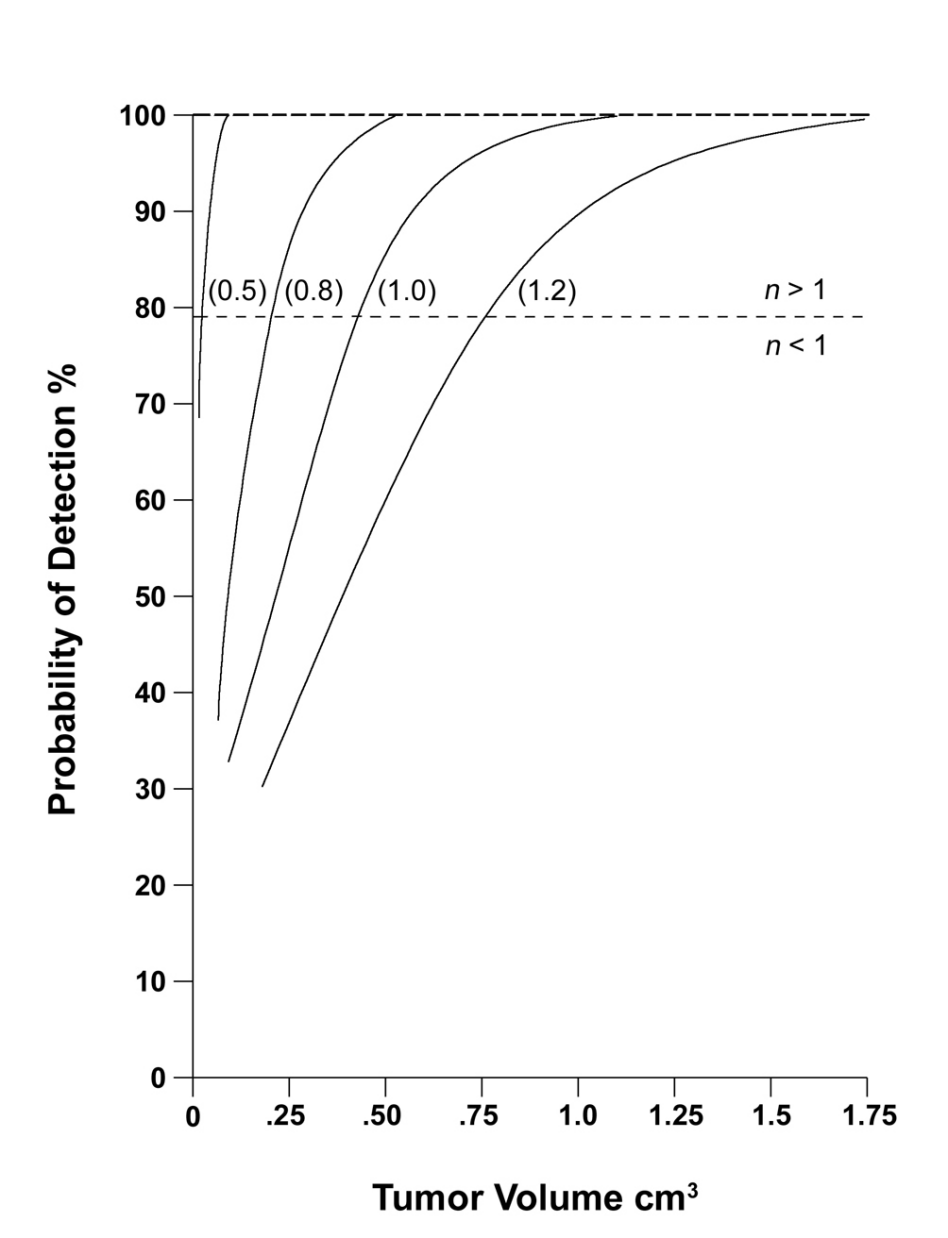
**Probability of detection versus tumor volume for different spacings of the finite core centers, from *S*= 0.5 cm to *S*= 1.2 cm**. The effective core spacing is *s *= *S *- *R*_*c *_in each case. for *n *< 1, see equation (1), and for *n *> 1, see equation (2). The dashed line at PoD = 78.5% identifies where *n *= 1.

Figure 4 is based on calculations using equations (1) and (2). It presents the relation between tumor volume, *V*_T_, and the probability of detection, PoD, for different finite core spacings. It demonstrates quantitatively the effect that decreasing core spacing has on increasing the probability of detection, at any tumor volume.

Figure 4 also shows the upper limits on tumor volume that could be missed, at different values of the spacing for the finite core center. For example, if core spacing *S *= 1 cm, then a tumor volume of *V*_T _= 1 cm^3 ^has a probability of detection that is greater than 99%.

**Figure 5:**
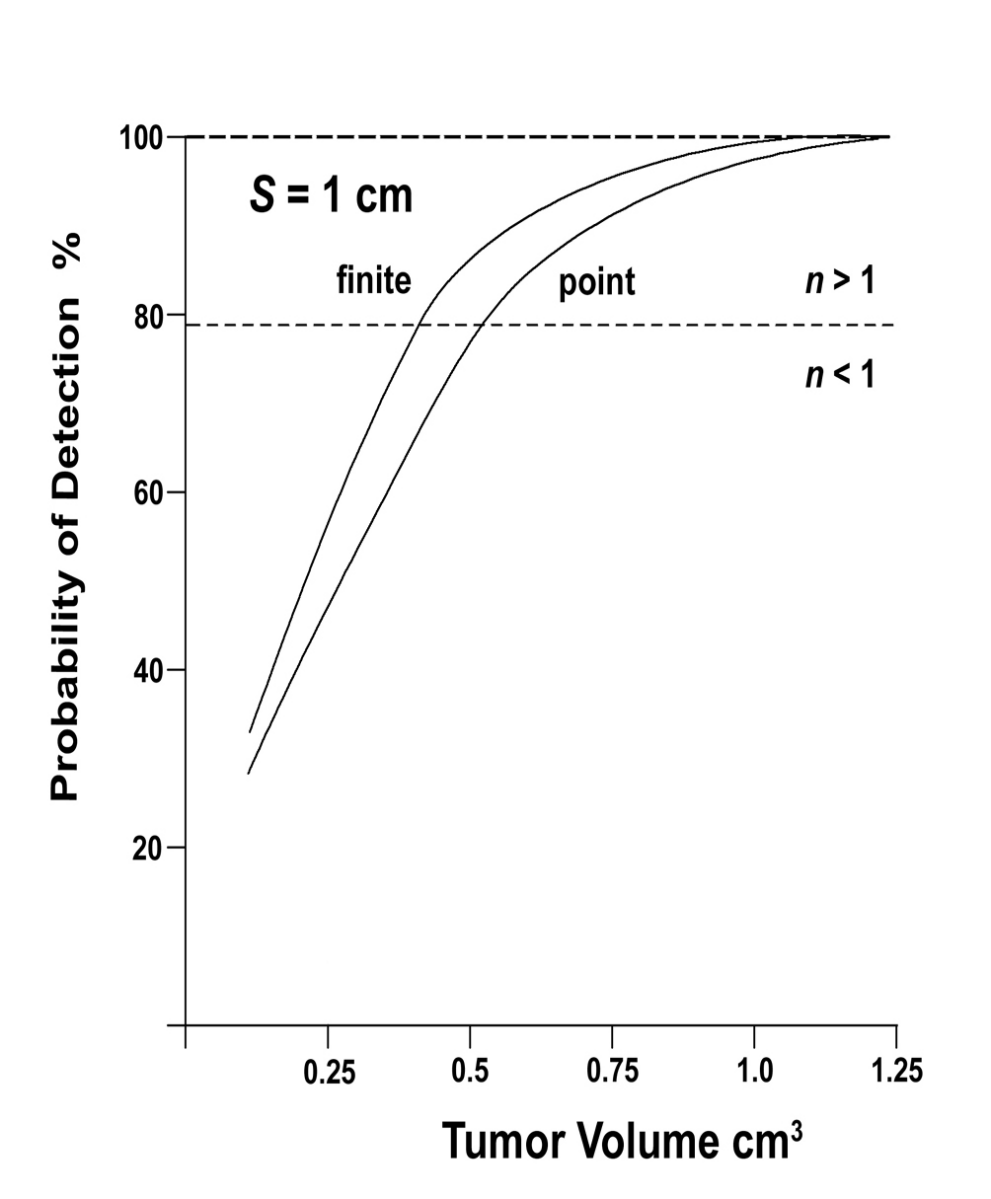
**Probability of detection versus tumor volume, comparing the point core and finite core cases, for *S *= 1.0 cm and *s *= 0.929 cm, using equations (A1) and (A7)**. The dashed line at PoD = 78.5% identifies where *n *= 1.

As developed in the Appendix, the finite core increases the probability of detection relative to the point core, at each tumor volume, see Figure 5. This effect is more pronounced as the core spacing, *S*, decreases. The fixed value of the finite core radius, *R*_c_, is increasing relative to the decreasing value of the core spacing.

**Figure 6:**
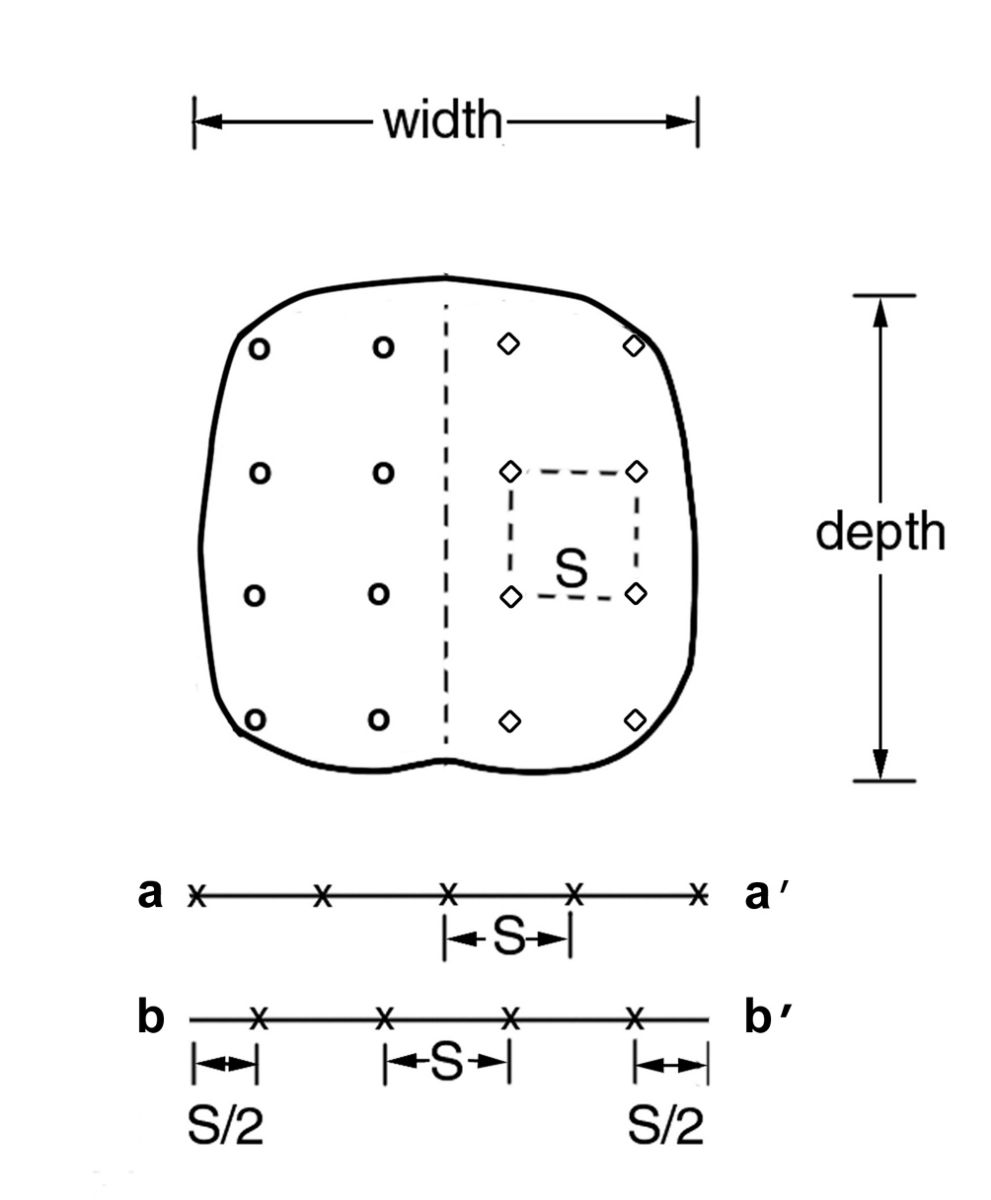
**Uniform grid for cores spaced *S *cm apart, with a transverse-plane section approximating an enlarged prostate, shown centered on the midline**. Open circles represent initial biopsy cores. Open squares represent repeat biopsy cores. Lines aa' and bb' illustrate the edge effect.

A grid of evenly-spaced cores is shown in Figure 6, with an enlarged prostate superimposed on the grid. For a given core spacing, a larger prostate will require more cores than a smaller prostate. The edge effect means that a biopsy core need not be placed closer than *S*/2 from the edge. Lines aa' and bb' (Figure 6) illustrate how the edge effect reduces, by one, the number of cores needed. The effect can also reduce the length to be sampled, by stopping short of the edge of the base. Thus, while extending biopsy core sampling close to boundaries is useful, it is not essential to come closer than *S*/2 to the boundary for the purpose of this analysis.

Bearing in mind the edge effect, an initial estimate of the number of cores needed is given by (*N*_c_)_est _= (transverse width/*S*) (transverse depth/*S*), see Figure 6. This estimate is refined by determining which (if any) of the grid points will require two cores stacked end to end along the apex-to-base sampling length. For example, let *S *= 1.2 cm, and consider a prostate with transverse width and depth both equal to 4.8 cm (this corresponds to the enlarged prostate shown in Figure 6). In this case (*N*_c_)_est _= (4.8/1.2) ( 4.8/1.2) = 16 cores. Assume only the eight grid points closest to the midline would each need two end to end cores. Therefore, the number of cores needed is *N*_c _= 16 + 8 = 24. In practice, adjustments based on the ultrasound-measured dimensions and actual shape of the prostate will be needed to establish *N*_c_. For the largest prostates, increased values of *S *will be required to keep *N*_c _at a manageable number. There is a basic trade-off with increasing *S*. It reduces (*N*_c_)_est _by 1/*S*^2^, but increases undetected tumor volume as *S*^3^. This is a strong incentive for making *S *as small as practicable, for the given prostate volume.

The approach developed here for distributing biopsy cores combines the available cores from an initial transperineal biopsy with those that could be available for a transperineal rebiopsy; call this total number of cores *N*_t_. Thus, one can plan in advance for distributing these cores, using the evenly spaced grid pattern, throughout the entire prostate--but using just *N*_t_/2 cores at each biopsy. Assume that no significant difference in tumor distribution appears on either side of the midline [11-13]. Using evenly spaced cores, place *N*_t_/2 of the available cores into one side of the midline at the initial biopsy (see Figure 6). If negative, place *N*_t_/2 cores into the other side at the rebiopsy. The advantages of this approach to distributing the *N*_t _cores are developed in the Discussion.

## Discussion

The ability to extract, via direct mathematical analysis, quantitative information on potential tumor volumes from a transperineal biopsy that gives a negative result expands the clinical utility of the transperineal approach--when used with an evenly spaced sampling grid of parallel cores. Increasing interest in this approach is leading to improved techniques and the recognition that it can provide thorough sampling of the entire prostate [14-18]. Computer simulation studies are also contributing new insights into the transperineal technique [11,19].

This quantitative information would be relevant to any clinical decision about what to do (watchful waiting, rebiopsy, intervention) following such biopsies. Figure 4 gave the probability of detection for a tumor volume, at different core spacings, when biopsies are negative. It offers a quantitative tool to help determine the template spacing options for placing the cores in a template-guided transperineal biopsy, and the number of cores needed, vis-a-vis the probability of detecting (or excluding) what one considers to be a clinically relevant tumor volume. It also demonstrates the increased efficiency of individual cores, when used in a uniform distribution pattern.

The relation involving tumor volume, core spacing, and the probability of detection is complex. This analysis leads to equations that quantify this relation and to Figure 4, which illustrates it in a practical way. Note that the analysis does not apply in the case of a positive biopsy. We are unaware of any work that provides an analysis of tumor volume for a positive biopsy, where now the primary clinical consideration becomes the Gleason grade of the tumor sample.

This approach differs from current studies aimed at using imaging techniques and sophisticated algorithms to locate, and identify positively, tumor sites. Such studies, in some instances, also attempt to estimate tumor volume. Current views suggest the need for further study to establish their clinical utility. Similarly, this paper seeks to motivate researchers to consider the advantages offered by this theoretical model for template-guided transperineal biopsies and develop their technique to test it.

The approach developed here for distributing biopsy cores overcomes problems with the systematic random biopsy approach, where the cores distributed throughout the prostate do not sample equal-sized regions--producing undersampling and oversampling. This reduces the detection efficiency of each core and increases, especially, the chances of missing a large tumor. Additionally, if a rebiopsy is needed, it is difficult to identify where the initial biopsy cores were taken throughout the entire prostate, again leading to undersampling and oversampling with reduced efficiency per core for the rebiopsy cores [10]. Our view of the biopsy protocol literature is that there is little consensus about the number and placement pattern of cores. The evenly spaced cores maximize each core's detection efficiency. Assume the initial biopsy cores, which were placed evenly on one side of the midline, are negative (no tumor detected). One then places all the rebiopsy cores evenly on the other side of the midline (Figure 6). This concept, by itself, holds equally well whether doing transperineal or transrectal biopsies. There have been no studies comparing the random to the uniform biopsy core pattern.

Biopsy technique also needs to focus on accurate template-guidance and the three dimensional approach because "...cores arrayed in three dimensions are superior to randomly distributed cores for detecting cancer." [5]

A uniform transperineal biopsy core grid pattern, as described here, has yet to be implemented. The mathematical analysis presented in this paper shows how to extend the usefulness of such biopsies, when negative, by providing quantitative data on the potential tumor volume that could be present. Assuming that the small tumors are spherical is a possible limitation, though common in theoretical modeling [2-4]. The transperineal biopsy technique requires adaptations to make use of the approach described here, such as the technical facility to place two cores stacked end to end that can sample adequately the apex-to-base distance, in larger prostates. Developing longer effective cutting lengths for biopsy needles would be helpful. Further development of template grid technology and magnetic resonance guiding for this biopsy approach is needed, to provide accurate three-dimensional prostate imaging along with reproducible guidance and tracking of the biopsy needles. This could entail the use of a robotic device to control the direction and uniformity of needle placement, as well as limiting needle deflection problems that can affect the ability to produce parallel cores, as assumed in the model [15-18,20-24].

The use of evenly spaced cores leads to a quantitative definition for the concept of saturation biopsy [25,26]. Saturation is defined by the value of *S *used in these biopsies. The lower this value, the higher the saturation. Thus, *S *is a singular measure of saturation that incorporates *both *the number of cores used and the prostate volume. With the transperineal biopsy approach, the evenly spaced cores can be located accurately with reference to the apex as the origin of a three-dimensional coordinate system [16]. This offers the possibility of unique comprehensive cancer mapping and facilitates comparative analysis of tumor detection data obtained from various sources and prostates [27-29].

## Conclusions

Each feature of this transperineal biopsy approach--the use of evenly spaced parallel cores, and sampling on one side of the midline initially--offers advantages for improving the ability of prostate biopsies to detect tumor and to extract useful data, on the potential volume of an undetected tumor, from a negative biopsy.

## Appendix

This analysis starts by asking, what is the largest spherical tumor volume that could fit between the cores (Figures 1A, B), and so go undetected at the transperineal biopsy? Conversely, the tumor volume that could always be detected is therefore only marginally larger than this largest undetected tumor volume. Thus, within limits inherent in this analysis, these volumes are virtually the same, for practical purposes.

The case of point cores will be analyzed and compared to the case of finite cores, with biopsy needle radius of *R*_c_. The point core case (Figure 2A) has a detection square with sides *S *cm. The inscribed circle tumor, and smaller tumors, are completely within this detection square. Tumors with larger diameters, up to the diameter of the circumscribed circle, are not completely within the square. Each of these conditions requires a different equation for calculating the Probability of Detection (PoD).

In the case of the finite cores (Figure 3A), the detection square is reduced by the finite core. The effective core spacing parameter becomes *s *= *S *- *R*_c_. The equations for the finite core case are the same as for the point core case, with *S *replaced by *s*. Figure 3A shows the tumor circle that just touches the inner edge of the four finite cores is positioned exactly at the center of the square grid formed by these four points of contact. In this position, it would go undetected. At virtually any other placement, it intersects at least one core and is very likely to be detected. This circle is defined, for the purposes of this analysis, as the smallest tumor volume that will have an effective PoD of 1.0. The analysis will show that even somewhat smaller volumes can have PoD values ≥ 0.99, and therefore are virtually certain to be detected. The tumor shown in Figure 3A has diameter *D*_T _= *S *- 2 *R*_c_. Any tumor with a greater diameter will be detected.

Consider the inscribed tumor circle (Figure 2A). The quarter-cylinder detection volumes (Figures 1A, B), do not overlap and the PoD is given by(A1)

The numerator is the volume of the four quarter-cylinders, i.e., equivalent to one cylinder of diameter, *D*_T_, and height, *h*. The denominator is the volume of a rectangular block with base, *S*^2^, and height, *h*. Thus, when *D*_T _= *S*, the PoD = 0.785. For this case, *n *= *D*_T_/*S*≤ 1.

When *D*_T _>*S*, the quarter-cylinder detection volumes will partially overlap one another. As shown above, *h *cancels. This reduces the problem to a two-dimensional calculation involving just the relative areas. Figure 2B depicts a quadrant of the total detection area, *S*^2^, for one point core--in terms of a tumor radius, *D*_T_/2. The PoD is calculated from the ratio of that part of the core's detection area (2 *A*_1 _+ *A*_sec_) that actually overlaps with the quadrant area (*S*/2)^2^. Set the ratio of the key parameters *D*_T_/*S *= *n*, where 1.0 ≤ *n *≤ , to simplify the calculation. Define the probability of detection as(A2)

given(A3)(A4)(A5)(A6)

therefore(A7)

given(A8)(A9)

so(A10)

For the finite core case, replace *S *with *s *≡ *S *- *R*_c _(see Figure 3B).

The steps in the calculation when *n *≥ 1.0 are:

1. Choose *S*, in cm.

2. Set *R*_c _= 0.05 cm.

3. Calculate *s *= *S *- *R*_c_.

4. Choose a range of values for *V*_T _and calculate the corresponding *D*_T_.

5. Calculate *n *= *D*_T_/*S*, or *D*_T_/*s*.

6. Using the value of *n*, calculate sin (Θ/2) and obtain Θ°.

7. Using *n *and Θ°, calculate the Probability of Detection from equation (A7), for each tumor volume.

Figure 5 shows that the finite core increases PoD, at a given tumor volume, versus the point core. For *n *≤ 1, equation (A1) becomes(A11)

Thus, for *S *= 1.0 cm and *s *= 0.929 cm, this ratio is 1.16. Whereas, for *S *= 0.8 cm, the ratio is 1.20. Therefore, for *n *≤ 1, as *S *decreases, this ratio increases. As expected, the finite core increases PoD, relative to the point core. For *n *> 1, this is also the case. However, as PoD values approach 100%, the probability of detection converges (see Figure 5).

## Competing interests

The authors declare that they have no competing interests.

## Authors' contributions

Both authors made contributions to each aspect of the paper. Both authors have read and approved the paper.
